# Doing Contrariness: Therapeutic Talk-In-Interaction in a Single Therapy Session With a Traumatized Child

**DOI:** 10.3389/fpsyg.2021.545966

**Published:** 2021-04-30

**Authors:** Michael B. Buchholz, Timo Buchholz, Barbara Wülfing

**Affiliations:** ^1^International Psychoanalytic University Berlin, Berlin, Germany; ^2^Freie Universität Berlin, Berlin, Germany; ^3^Independent Researcher, Cologne, Germany

**Keywords:** trauma, children in treatment, doing contrariness, psychotherapy, adoptive children, conversation analysis

## Abstract

Conversation analysis (CA) of children-adult—interaction in various contexts has become an established field of research. However, *child therapy* has received limited attention in CA. In child therapy, the general psychotherapeutic practice of achieving empathy faces particular challenges. In relation to this, our contribution sets out three issues for investigation and analysis: the first one is that practices of achieving empathy must be preceded by efforts aiming to establish which kind of individualized conversation works with this child (Midgley, 2006). Psychotherapy process researchers in adult therapy (Stiles et al., 2015) have found that therapists “invent” a new therapy for each patient (Norcross and Wampold, 2018). The second issue is that it can be difficult for adults to understand the ways in which children express their conflicts and issues. In particular, play activities in therapy, e.g., with dolls, can open up additional scenarios of interaction. The play scenario can be used to disclose unformulated problems masked in everyday and family interactions. The third issue is how to respect the child's higher degree of vulnerability, compared with adult patients. How is it communicated and dealt with in therapy? We present an interaction analysis of a single case study of the first 20 min of a child therapy session with an adopted girl aged 4 years brought to treatment because of “unexplainable rage.” The session was videotaped; parents granted permission. We analyze this session using an applied version of CA. In our analysis, we describe “doing contrariness,” as a conversational practice producing epistemic and affiliative disruptions, while “avoiding doing contrariness” and “remedying contrariness” are strategies for preserving or restoring the affiliative dimension of a relationship (in child therapy). We show how these practices operate in various modes and how they are used by both parties in our case study to variously aid and impedethe achievement of empathy and understanding.

## Introduction: Doing Contrariness

Interaction involving children has been extensively studied in conversation analysis (CA) (Goodwin, 2006; Kidwell, 2013). CA has studied treatments of children with language disorders like stuttering (Leahy, 2004; Leahy and Walsh, 2010) and children with autism spectrum disorder in an educational environment (Korkiakangas et al., 2012) and in parental care (Ramey and Rae, 2015). Others have studied interaction with children with Asperger's syndrome (Rendle-Short, 2017), the role of fatherhood in family therapy (Suoninen and Wahlström, 2010), or of morality in helping institutions (Bergmann, 1990).

CA studies of the entire process of child *therapy* have been conducted (Gardner and Forrester, 2010; Lester and O'Reilly, 2015; Stafford and Karim, 2015; Rendle-Short, 2017) to study the influences of parental behavior, of institutional contexts like schools or psychiatric wards. Studies contrasting CA perspectives on interaction with theory-of-mind approaches have documented the influential contribution of the helping personnel like psychiatrists or teachers or even parents, in producing a child's symptoms (McCabe et al., 2004; Stivers et al., 2017).

Hutchby (2007) studied the work of counselors with children of 6 years (or older) whose parents were divorcing. The counselors' participation has the aim of helping these children to better cope with their parents' divorce. Counselors bring to the consultation the success of therapeutic vision, in seeking to bring into play counseling relevant topics and interpretations, but achieving their aim depends “in part on children's recognition of, and willingness to go along with, that aim” (p. 131). Hutchby documents that handbooks for counselors do not sufficiently inform on how to achieve that aim because they ignore the conversational details. He makes a strong argument for studying the “true richness of the interactional resources brought into play by both counselors and children” (p. 134). This “true richness” he calls *bricolage*, a kind of do-it-yourself for the counselor. S/he has to invent therapy based on skillfully taking the child's individuality into account. However, the aim—to help with coping with the parent's divorce—is predefined by the institution. The counselor's skills aim at winning the child's participation, but he crucially notes that children often resist; this is most impressively shown *via* the case of a boy of 6 years responding to the counselor's questions with “I don't know” more than 60 times in a single session. Hutchby's analysis shows how “don't know” is not merely an assertion about insufficient access to or lack of information. The response is identified as part of a “game” played with or against the counselor, or even as a strategy (p. 117). Hutchby here uses the term “resistance” as equal to “unwillingness” (p. 121); in medical sociology, the term “non-compliance” is used to describe when patients do not follow their doctor's instructions.

Hutchby's findings on child *counseling* can be fruitfully compared with the situation of child *therapy*. The child counseling described by Hutchby has a predefined target: learning difficulties, parental divorce, bullying at school, etc. It is also time limited (5–10 sessions) and mostly offered in an institutional context. The kind of child therapy we describe here often lasts many more sessions and frequently takes place in private practice. It also often has no institutionally predefined target. Instead, as in our case study, it is frequently the parents who seek to predefine the aim of therapy, and therapists may fear that the child will be taken out if the therapy does not conform to these aims. Young children do not openly articulate self-defined aims for therapy in many cases. Therapists try to find out about their aims by observing how the child comments on being brought to therapy. Therapists try to “read” these comments, observe the child's play for references to an unformulated grievance, unrecognized pain or conflict, or use other observables to decide on how to *individually* perform therapy with a child. If things go well, it can happen that the child likes coming to therapy and that this can lead to her/him setting her/himself up in a somewhat opposing position to that of the parents. This can manifest in practices of what we call “doing contrariness” (DC). We will discuss instances of DC as they appear in the analysis and only give a short characterization here. It is a conversational practice involving a one-sided use of power by one of the participants which thwarts another person's plans or expectations. The use of power consists in creating a distinction, and assuming the authority of valuing one side of it as positive, the other as negative. Its operational mode is often as a disruption, violating expectations. Because of its disruptiveness, it can have considerable effects on participants. Both the conversational disruption that it consists and the unbalanced distribution of strongly positive and negative emotional values that it affects can threaten their affiliative network. We also observe practices responding to or preemptively seeking to avoid contrariness, aimed at restoring conversation and affiliation or at keeping them intact. These repair activities (in a broad sense) we call “remedying contrariness” (RDC) and “avoiding contrariness” (ADC), respectively.

## Materials and Methods

We provide an interactional analysis of a therapy session with a young child. Ina (pseudonym) is a 4-year-old girl. After giving birth to her, her mother left her in the care of a hospital. Ina was placed with a foster care family for half a year and then was adopted. She is brought to treatment because of frequent outbreaks of rage against other children and her adoptive parents. The clinical hypotheses for her are that she has a deep desire for her mother's love and in the same moment a strong hatred toward her for having left her alone. This is a strong emotional burden and a cognitive conundrum she cannot solve. A further clinical hypothesis is: parents in adoptive families often wish to be accepted as “genuine parents,” addressed as “mom” and “dad.” After some time, adoptive children often realize what a powerful position in family life they can achieve by *not* fulfilling their parent's wishes (Feder, 1974; Nickman, 1985; Haimes, 1987)—a generalized example of “doing contrariness.” Ina's situation, as a child having suffered from traumatic losses in early years requires a different therapeutic approach than the one documented in Hutchby (2007). The session has been recorded on video and audio. The parents have given their permission to use the material for publications.

### Our Study Aims at Answering the Following Guiding Questions

How are the conventions of conversation adapted to the purpose of therapeutic talk between therapist and child? Do they use playing scenarios to negotiate and solve conflicts arising from the traumatic history of the child, and if so, how? How is their conversation affected by whether they have aligning and non-aligning interactional goals? We focus on one interactional practice (doing contrariness) and strategies to address it (remedying/avoiding doing contrariness) which we identify in various multimodal guises and show which role they play in answering these questions as well as for the therapeutic process of treating a child with traumatic experiences. We hope to contribute to a better understanding of therapeutic interactions, which has potential benefits also for therapy: applied analyses such as the one we are presenting can have a kind of supervisory function for therapeutic talk and for institutional conditions (Karim, 2015). CA research has also contributed to a better understanding of the details of children's talk about traumatic experience (Bateman et al., 2015).

Although we are oriented toward CA in our analytical approach, we also make use of the toolbox of broader linguistic analysis as well as insights from psychotherapeutic research and clinical experience.

There are two main issues that are particularly important for our analysis of the interactional processes in child therapy and of how they influence therapy outcome, in our view. They concern the length of conversational stretches that are usually considered in analysis and the question of how to deal with less conventionalized language (here, of and toward young children).

Regarding the first issue, in CA, analyses are often conducted on sequences of only a few turns in length; this has produced the very influential concept of the adjacency pair (Schegloff and Sacks, 1973). Certain adjacency pairs in specialized contexts have not only produced highly conventionalized linguistic forms, such as greeting-greetings, but also various kinds of offers, requests, invitations, and their respective responses. There is a preference bias for the production of positive responses over negative ones, and in perception, negative responses are also dispreferred in that they cause a higher cognitive load (Bögels et al., 2015; Kendrick and Torreira, 2015), leading to conventionalization also in the type of response, not only its form. Sometimes, analytical attention in CA can seem to focus on short sequences and strict sequentiality. However, this is not so on principle: Sidnell (2013) discusses several types of evidence in CA that are not restricted in this way, and Schegloff (2007) substantially extends the notion of adjacency pair beyond immediate sequentiality *via* expansion. Recent pragmatic models also show the relevance of meaning dependencies across longer stretches of discourse in everyday conversation (Farkas and Bruce, 2010; Ginzburg, 2012; Roberts, 2012; Goodwin, 2015). Together, these approaches allow to take effects into consideration that arise from the interaction of the content and illocutionary force of the current turn with (the retrospective interpretation on behalf of the participants of) conversational acts having taken place many more turns before. This is particularly important for understanding conversation in a psychotherapeutic setting, where long-distance effects and their negotiation between therapist and patient are part and parcel of the interaction (Buchholz and Kächele, 2017). One of the aims of CA has been to contribute to an understanding of how meaning is “achieved” in interaction (Garfinkel, 1967; Heritage, 1984). The understanding of meaning underlying this idea is that it

…lies not with the speaker nor the addressee nor the utterance alone as many philosophical arguments have considered, but rather with the interactional past, current, and projected next moment. The meaning of an entire utterance is a complex, not well-understood, algorithm of these emergent, non-linear, sense-making interactions (Schegloff et al., 1996, p. 40).

Since we here are also interested in this interactional aspect of meaning as something that arises out of the pragmatic negotiation between therapist and patient, we also adopt a methodological stance that considers more than the immediate context of a turn in order to make this meaning visible. Focusing on a single session and proceeding chronologically over a relatively long part of it (roughly 20 min), our analysis shows how certain topics occurring at the beginning resurface at a later stage and are then negotiated again in the light of the progressing discourse. Themes from the previous day's session are also continued.

The second issue concerns unconventional language. In close adherence to ethnomethodology (Garfinkel, 1967), the action a turn performs can be interpreted in CA partially *via* the immediately ensuing response of the interlocutor, relatively independent of the formal linguistic content of the turn. That does not mean that the formal content is of no interest to CA analyses. As an example, if an interlocutor begins a conversation by saying, “I hate having to see your face again every morning,” and this is met by a response along the lines of “Yeah, good to see you too, Bob,” then by the principles of CA, the facts that these are the initial turns of a conversation and that the first turn is treated by the interlocutors as if it were a greeting, will make sure that such a sequence is correctly analyzed as a greeting-greeting pair, even though the first turn is a highly unconventional member of such a pair. Here, we are also interested in this unconventionality, and what effects it might have. While such an exchange might actually conform to the specialized greeting conventions established between a specific pair of coworkers, treating it as just any greeting-greeting would miss crucial aspects of the social actions performed by it, especially if it occurred in a therapeutic context. Heritage (2011a) has pointed out the risks of concentrating on conventional language in the context of medical interactions^1^. This is at least as appropriate in the case of psychotherapy. Although psychotherapeutic theory has developed just as much specialized jargon as any profession, in the conversation between patient and therapist, there is no predefined set of conventionalized terms one has to learn in order to participate successfully. Pawelczyk (2011) talks of “personalized meanings.” For each therapy, patient and therapist invent and locally conventionalize a set of terms and routines together. For the successful analysis of psychotherapeutic interaction, it is necessary to allow for the treatment of (in a global sense) unconventional linguistic and interactional behavior, including not only neologisms, creative imagery, and metaphors but also the omission or delay of expected responses and other forms of non-cooperative communication, as meaningful in its unconventionality. All of these forms of communicative behavior are means to expressing thought processes, intentions, and emotions which the interlocutors are not used to putting into words and for whose expression perhaps no conventionalized means exist, and which the goal of the therapy it is to negotiate and resolve in the personal interaction between therapist and patient. In child therapy, these challenges and difficulties are exacerbated many times. An individualized way of interaction has to be established between the interlocutors, only by means of which conversational meaning can be created. In the conversation we analyze, therapist and patient often ostensibly talk about some object in their surroundings, while at the same time they negotiate a personal issue of the patient in a therapeutically relevant way. In order to show that this is the case, we make use not only of CA methods but also of a broader range of pragmatic analysis as well as insights from clinical experience. However, our analysis is empirically anchored to the observables of the interaction. Psychotherapy talk-in-interaction is accessible to empirical methods beyond introspection.

We provide an interaction analysis of segments from the transcript of the first 20 min of a video-taped child therapy single session conducted in the private practice of the third author^2^. We present English translations of the German GAT-2 transcripts (Selting et al., 2011) including descriptions of the bodily behavior of both participants, additionally illustrated by pictures. The entire interaction was divided into segments that constitute therapeutically relevant episodes. We proceed chronologically through the segments, however omitting some of them for reasons of space. Those that are included were selected for showing how some therapeutically relevant development is related to instances of doing or resolving contrariness. For speech acoustic analyses of the same session, see Dreyer (2018). Other analyses with the same material (a qualitative multimethod project) have been published (Brandstetter, 2018; Dreyer, 2018; Hamburger and Bleimling, 2018; Heller, 2018) as part of a collaborative effort from our Berlin-based research network to throw a light on the complexity and variety of information contained in such material by illuminating it from the angles of various disciplines and approaches.

## The Beginning of the Therapeutic Session

In the first segment, directly from the beginning of the session, we witness how Ina uses conversational non-participation to do contrariness, with the effect of excluding her adopted father from the conversation. The session starts when Ina, just brought in by her adoptive father, puts her head around the door frame to look into the play therapy room:

### Segment 1—Introducing T, Therapist; F, Father, and Ina


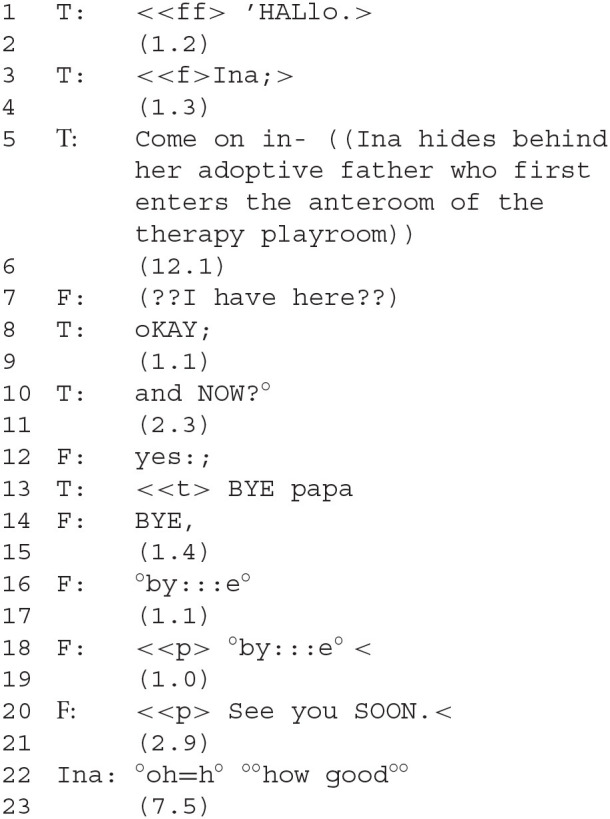


The therapist offers several greetings to Ina (1–5), who does not respond with a greeting of her own. This constitutes an act of refusing to submit to what Goffman termed “mutual monitoring”:

“Persons must sense that they are close enough to be perceived in whatever they are doing, including their experiencing of others, and close enough to be perceived in this sensing of being perceived.” (Goffman, 1963, p. 17)

That is just what the therapist attempts to initiate with Ina, after she has been brought in by her adoptive father. People take note of each other in greeting and give a conventionalized signal that they acknowledge the interaction as such. In adult life, greeting response is considered conditionally relevant (Sacks and Schegloff, 1979). A refused greeting can be experienced as a violation of expectations by the greeter and this can potentially be exploited by the non-greeter. A non-greeter does more than just refuse greeting: s/he denies “mutual monitoring” by purposefully violating expectations. Such an omission has been termed a “noticeable absence” by Sacks and Jefferson (1992/1995). Omitting the greeting can here also be considered an instance of “doing contrariness.”

Silently Ina takes off her coat (during a long pause, line 6). The therapist initiates the expectable process of saying good-bye to the father as a next step by articulating an expectation: “and NOW?” (line 10). After a pause the father responds with a second position utterance, his slightly extended “Yes” (12). This sequence seems to have no content. However, the situational context indicates that it is time to say good-bye to the father, so that Ina's session can begin. Both adults seem to expect Ina to initiate or at least to contribute to this procedure, none of which she does. Her continued conversational inactivity has strong effects on the “participation framework” (Goodwin, 2018) of the two adults: the therapist now assumes Ina's role and starts the farewell sequence (line 13), indicated by adding “papa” after her “BYE.” The therapist seems to “jump in” (Corrin, 2010): she must have deemed a contribution by Ina to be so necessary that not only did she take it upon herself to provide it, but she also performed it as if she were Ina herself. Ina's “doing contrariness” by non-participation in the practice of saying goodbye amounts to refusing to acknowledge the father's presence at all.

The father tries four times, with pauses in between, to get Ina to say goodbye (14–20)—but no response from Ina can be elicited although he uses a variety of prosodic contours and lexical expressions to say goodbye. His expectation to be responded to is not fulfilled, and yet he simply tries again and again, instead of explicitly addressing Ina's failure to conform to interactional expectations—we could call this “doing vulnerability,” as counterpart to Ina's “doing contrariness.” Both adults interpret Ina's “doing contrariness” as repairable *via* a silent but powerful communicative agreement to further *ignore* Ina's behavior. The therapist attempts to mitigate the affiliative damage caused by Ina's behavior by acting as a version of Ina that does comply with the adults' expectation. Their agreement is sealed by the father accepting to be vicariously addressed as “Papa” by the therapist—a rather unusual communication among adults. Father and therapist seem to agree to treat the therapists' response in Ina's stead as if it were sufficient “absence of evidence of misunderstanding” (Albert and Ruiter, 2018, p. 281) to be able to treat the entire episode as constituting successful communication.

A new participation framework between Ina and the therapist is eventually established by Ina's first remark, “how good,” after the father leaves. That she speaks for the first time only once he leaves, and gives a positive evaluation directly upon his departure indicates that her “doing contrariness” was directed against the father. She is willing to participate but only once he is gone.

We have seen in this first segment how “doing contrariness” (DC) is characterized by behavior that violates conversational expectations and uses this violation to affect the affiliation and emotional ties between participants. Fundamentally, DC makes a contrastive distinction, valuing one side as positive, the other as negative. In this first segment, it is the adoptive father as participant in the interaction who is valued negatively by Ina's refusal to participate until he is gone. Both the act(s) by which DC can be performed and its effects are highly contingent upon the existing affiliative network between the participants and the roles they take on in the conversation: if it had been the therapist who had performed a silent non-participation, this would certainly not have affected the father in the same way. Attempts at repairing DC (which we call “remedying contrariness”) must be equally sensitive to the participants' network. The therapist's “jumping in” for Ina, and the father's going along with it, can be seen in this light. This is an action which only repairs the conversational dimension of the disruption Ina has produced in the sense that it at least allows the conversation to proceed in the direction projected by the father and the therapist. The therapist-as-Ina and the father use this role performance to eventually move on beyond Ina's non-participation, but by allowing his multiple attempts at saying goodbye to remain unreciprocated, the father shows that the affiliative damage is left unrepaired. However, under the circumstances, it might have been the least damaging course of action overall. Directly addressing Ina's non-participation and asking her to “correct” it would probably have caused further affiliative damage. Executing a correction implies that the preceding move is faulty or problematic in some aspect, and thus that the sequence is marked/unexpected from the perspective of a smooth discourse progression (Ginzburg et al., 2014; Łupkowski and Ginzburg, 2016). Between adult speakers, and executed without face-saving actions, a correction would additionally violate conventional social expectations and thus threaten the affiliation between participants. It would be a claim to authority by the correcting speaker and reduce the other speaker's agency. It would thus also be what we call doing contrariness. The therapist (or the father) could have presented Ina's non-participation as such a disruption by attempting to “correct” it with a direct request toward her to participate. This might have “repaired” the conversation by forcing her to contribute, but given Ina's demonstrated contrariness, it would have likely been taken as a rebuke and, instead of repairing the affiliational disruption with Ina, would have instead exacerbated it. Foregoing the opportunity to assert their own deontic authority and power and to incurring some face loss themselves, the therapist and her father spare Ina the face-impairing and emotionally hurtful act of correction. ADC can therefore be distinguished from other repairs by targeting not only the communicative disruption but also the social-affiliative disruption caused by DC; it cannot be performed *via* an act of DC itself. The type of creative solution the therapist and father use to try to avoid the incurrence of further affiliation cost is often what is needed in psychotherapy in various modes and situations, and one of the reasons it can be so difficult.

“Doing repair,” especially self-repair, can help to maintain or improve social relationships (Schegloff, 1992). After one party has violated the other's expectation, repair acknowledges a failure in performing a relevant contribution and accepts the obligation to an undisturbed common sociality. The crucial aspect here consists of acknowledging the other's expectations. In Schegloff's words:

“This is the sense in which these repair positions provide a defense of intersubjectivity. They are the last structurally provided positions because after these positions there is no systematic provision for catching divergent understandings.” (Schegloff, 1992, p. 1325)

Young children in medical consultations have often been observed to not respond to greetings (Cahill, 2010). In our case, several attempts at repair do not succeed and leave the affiliation partly damaged. Our analysis of this DC *performed through silence* shows that the new framework between therapist and Ina has a “history” burdened with this failed repair.

In the following segment, we will further explore the crucial dependence of DC on the role that a participant takes on in a conversation, in that we will see it creating a negative evaluation of part of one's self.

## The Creation of a Common (Play) World

### Segment 2—Starting Therapeutic Conversation


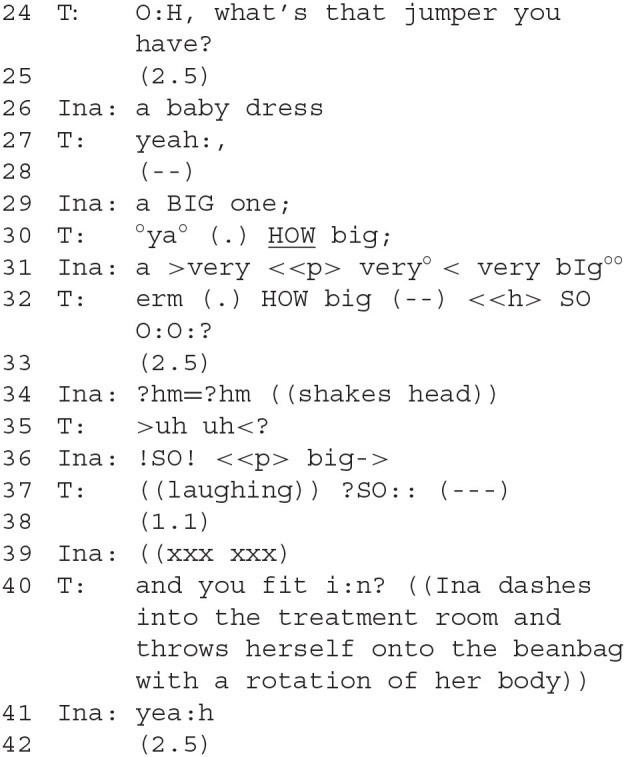


The therapist's question (24), prefaced with a playful interjection of surprise and amazement, is accompanied by a deictic gesture; *via* her gaze, she points at Ina's jumper. Slightly disparagingly, Ina calls it a baby dress (German “Babykleid”) (26). This is contrasted by her subsequent assertion of the “bigness” of the jumper (29).

The therapist initiates the next sequence of questions, “how big?” (30 f.). She suggests a size *via* gesture (32) and Ina rejects the suggestion *via* a shaking of her head and a negative interjection (34). The following rhythmical copy of that interjection by the therapist uttered with question intonation (35) resonates at the affiliative level, while also conveying amazement and surprise. The epistemic question of how big the sweater is, repeated in abbreviated form, is replied to by Ina's embodied demonstration (36), responded to by the therapist's surprised response (37) again with a gestural demonstration of size. After a pause (38), suggesting the topic has closed, the therapist takes the topic up again: “And you fit in?” (40), explicitly establishing the link between the jumper's size and Ina's. Ina's strong embodied response (40) demonstrates her body control and strength, signaling that she is already big. Only then follows her spoken confirmation (41). Ina clearly shows a very different attitude to the attribute of “bigness” than to that of baby-like qualities from the beginning of the segment.

Moving to a more therapeutic aspect of the analysis, Ina's disparaging voice when talking about the “baby dress” indicates a distance to the “baby-self” in favor of a more grown-up “child-self,” which is as “big” as the jumper. Talking about the size of the jumper implicitly has a bearing on Ina's status as either baby or older child. The jumper is used for a comparative contrast between two available selves, a “big” one and a baby one.

Ina clearly positions herself as being “big,” and expresses a negative evaluation toward being “a baby.” There are observable indications (disparaging prosody when discussing the “baby dress,” augmentative repetition and emphatic prosody when discussing the “bigness,” the explicit link made by the therapist between the sweater and Ina, agreed to by her) that the conversation is not only about the sweater, but also between two versions of Ina in play here. Ina distances herself from a version of herself that is “small.” While children occasionally express wishes to be more grown up, in this specific case, we take this to be a clinically relevant observation, namely as an indication of a strong negative affect toward her own (past) self, probably related to her traumatic adoption experience. In this section, DC is mostly visible in the negative attitude Ina expresses toward her “baby-self.” The contrariness that is being done, the hostile attitude Ina expresses toward herself and the repair efforts undertaken against it is best analyzed from a perspective which understands that Ina can act as either of these roles, the “baby-self” and a “child-self,” that are in conflict with each other. We argue that this is an example for how therapeutic observations can work together with CA in an understanding of what happens in therapeutic conversation. An analysis of therapeutic conversation must be able to capture such aspects in order to establish a connection between interactional observation and clinical interpretation and outcome. We suggest that there exists a need in the analysis of child therapeutic conversation both for special attention to unconventional means of creating pragmatic meaning (talking about the sweater as a stand-in for talking about Ina) *and* for observing long-distance relationships within the discourse progression.

## Building The Game—A Baby is Made

Conversational events and actions diverging from expectations have high *epistemic* (“novelty”) value because they have the potential to increase knowledge by adding unexpected (i.e., new) information. However, they bear the risk of causing affiliative disruptions, especially when concerning issues that the participants are emotionally invested in. For therapeutic practice, this means a delicate balancing task: therapists aim at exploring expectation discrepancies not as disappointments (= violating their own *affiliative* expectations in order to achieve a high epistemic value) but instead work to combine their high informativity with an effort at restoring the affiliative dimension at the same time. We have seen an attempt at such an other-oriented affiliative repair already when the therapist stood in for Ina saying goodbye to her father. In the following segment, we will see how such an repair attempt can also be self-oriented to prevent a previous action from becoming a disruption and damaging affiliation, literally seeking to *avoid* doing contrariness. Such a task includes transforming the therapist's own *affiliative* disappointments (and other affects) into something that is *epistemically* relevant. We will see how such an attempt can fail.

### Segment 3—The Table Scenario Is Prepared


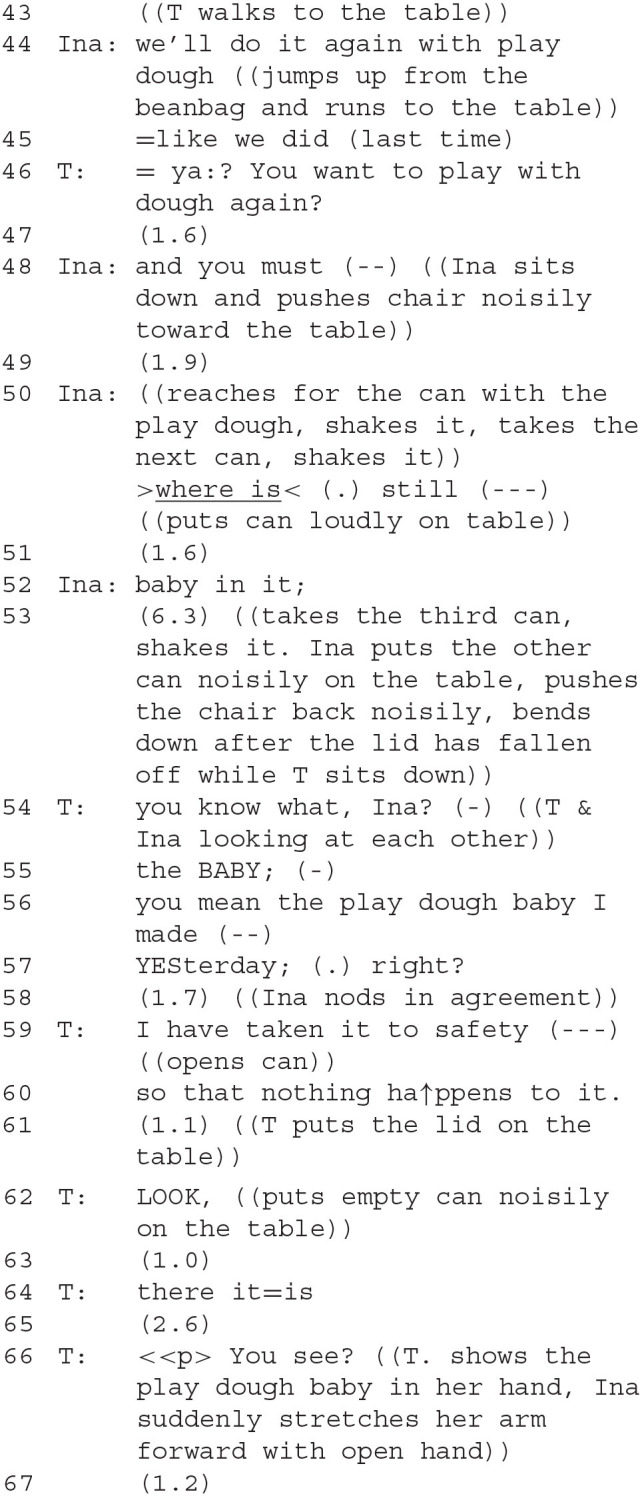


Both move to play at the table. Ina indicates continuation of a play project from the past session (43). The therapist reformulates Ina's project (45), yet in a slightly different way. Ina declares that she wants to do “it” again with play dough (German “wir machen *das* wieder mit Knete”), but the therapist asks for confirmation about playing with the dough. It seems that for Ina, playdough is not the actual object of play, but a tool for another play activity. Her non-response to the therapist's clarification question is again a “noticeable absence,” indicating a contrast between the two projects, or perhaps an objection to the therapist's question.

Ina tries to assign play roles (48), but her deontic authority is limited; now, it is the therapist who does not respond. Another obstacle materializes: Ina cannot find the playing equipment where she expected it to be (49–52). Noisily, she searches for the play dough baby, without success.

The therapist, after silently observing Ina's activities, starts an account (53), prefaced by “you know what, Ina?,” responded to by an intense exchange of gazes. Then follows another preface after a self-repair—the turn construction is interrupted (54) by a reference to what Ina did (55) and a confirmation that the therapist understood what Ina was looking for, with a projected ending with a tag (56), to which Ina agrees by nodding. However, she crosses her arms, a gesture expressing a mood between expectation and defiance. By using several prefacing moves, the therapist attempts to *avoid* doing DC herself, because she probably suspects that her intention to inform Ina that the baby is “taken to safety” (58) is likely to become troublesome, since Ina is already signaling some discontent.

However, she cannot fully avoid DC here. By opening the can, presenting the playdough baby with “there it is” (63) and “you see” (65), the contrariness is exacerbated: Ina is informed that she was searching in vain while the therapist knew where the baby is, that the therapist did not cooperate in her project. The affiliation framework is at risk, Ina's disappointment might transform into defiance. The therapist attempts to restore a “common ground” by presenting the play dough baby, efforts designed at balancing their epistemic positions by sharing knowledge with her that had been privileged before.

The therapist clearly indicates that she appreciates the complexity of this interaction. She cannot entirely avoid doing contrariness directed at Ina, in the sense of taking an opposing position, because her actions of hiding the figure and only disclosing its location at her own discretion reveal that while Ina might make claims to authority, it is the therapist whose deontic (protecting the doll) and epistemic (determining and knowing its location) authority is actually superior. She tries to mitigate the force of this contrariness *via* special conversational means, by formulating cautiously, using tags like “right?,” and by integrating Ina's perspective with phrases like “you mean…,” “look” and “see.” In this, she treats Ina as if Ina's and her own claims to authority (epistemic and deontic) were actually balanced, i.e., she affords Ina some authority as if she were an equal, e.g., another adult (Hagemann, 2009; Jefferson, 2012). Her hedges all gently ask for Ina's agreement to the goal that the baby figure should be protected, that in fact the therapist's project, while seemingly contrasted with Ina's, actually aligns with her interests too. In this way, she attempts to convey the epistemic content (the act of revealing the figure's location and her motives for hiding it) without incurring affiliative costs, and to point out ways of conversational negotiation that do not have to involve all the negative effects of “doing contrariness.” Ina makes no indication of giving this agreement.

We suggest that the therapist's effort at *not* doing contrariness has parallels in other aspects of human interaction. Hutchby describes how the child in his case uses “I don't know” sometimes as a game or a strategy. He adds:

“However, at certain points in the child's talk we find evidence that, for him, answering with ‘Don't know' is itself a way of producing serious talk. In other words, the child occasionally uses ‘Don't know' in such a way as to display that he is not playing a game.” (Hutchby, 2007, p. 115)

“Not doing something” has been earlier observed (Bateson, 1981) in how dogs distinguish between “playing attack” and seriously biting. Dogs (and certainly humans) can perform actions to demonstrate that they are *not* doing a different action that might also have been executed in the same context. By this process of implicature (Grice, 1989; Levinson, 2000), the *contrast* itself between one action performed to another *not* performed can be used as a resource to continue conversation, and to keep it e.g., on the side of playfulness (in the case of the dogs). In this segment, we have seen how the therapist used hedges and integrating formulations instead of doing contrariness, i.e., as actions that are performed partly because relevant alternative actions would have involved doing contrariness (with all of its disruptive effects). This was done in order to not violate the affiliative dimension of her relationship to Ina (or repair such a violation). We call this “*avoiding* doing contrariness” (ADC). Our inspiration for that comes from a remark by Tarplee (1996), observing how mothers of 2-year-old children teach them the pronunciation of difficult words, after the child has made an initial mispronunciation:

“The way they come off is not as corrections, but as re-elicitations. By *avoiding* doing contrastivity, and by being delayed, they appear to ‘try again’ - to give the child an opportunity to have another go - without explicitly indicating that the child's first attempt was problematic. In this way, they seem to manage the work of repair in a particularly subtle fashion.” (Tarplee, 1996, p. 426) (italics in the original).

Tarplee notes that “contrastivity” is *avoided* by the teaching adult *via* delaying an utterance which corrects the child's failed attempt. Repeating the word without delay would signal the child's attempt as having been “problematic,” in the manner of an other-oriented repair; but by delaying the repairing utterance, it becomes ambiguous between a response and a renewed initiating move, a prompt for the child to have another go. The too obvious contrast between the child's and the adult's pronunciation is made less prominent. *Avoiding* some of its impact has implications for the epistemic and deontic hierarchies between the speakers. Tarplee is mainly interested in the prosody of correction and in showing that already children of that age are capable of recognizing it; her “contrastivity” is quite closely related to the general notion of “contrast” between alternatives (here, the differing pronunciations) in linguistic pragmatics. We take her observations only as a point of departure and would like to concentrate on another aspect here: spacing the utterances apart in time and thus avoiding the contrastivity means the mother is effectively treating the child like she would an adult, hedging a correcting move in order to not infringe upon the other's epistemic or deontic authority. Producing the correction in direct temporal succession to the child's attempt would be to exploit the actually existing difference in deontic and epistemic authority between adult and child, but not doing that protects the child's vulnerability by affording her/him the authority s/he cannot claim for her/himself. The adult treats the child as if correcting her without hedging could be taken as “doing *contrariness*”: if the same interaction took place between adult equals it would likely be contrariness, because the unhedged correction could be taken as an unwarranted claim to authority and negatively affect their affiliation. Avoiding this action has the potential of raising the child's status above that which she is conventionally entitled to and thus strengthening her affiliation with her. We observed something similar in this segment: the therapist attempted to downplay the imbalance between her own epistemic and deontic authority and that of Ina, trying to avoid appearing to impose her authority. Therapeutically speaking, repairing instances of doing contrariness is highly important: unresolved episodes of it could turn into a lasting strain on the patient-therapist bond, constituting a rupture in the therapeutic alliance (Safran et al., 2011). In contrast to the instance of RDC seen in the first segment, which was a kind of other-oriented repair aimed at restoring affiliation, in this case, ADC is performed almost simultaneously to the action that is potentially doing contrariness, and it is self-directed.

## Summary of Intervening Segments

We leave out the next four segments (4–7) of the transcript (89 lines in the unabridged transcript^3^), but give a short summary, so that readers may still follow the development of the session. The therapist still attempts to negotiate a mitigating solution for the conflict that has just occurred, asking whether Ina wants to form her own play dough baby and offering to make one for her. Ina refuses and mostly responds bodily, kneading her fingers and reaching forwards as if to grasp the play dough figure. The disaffiliated context *seems* not fully repaired. Gradually, Ina's family history is integrated into the play scenario on the table in a form of reenactment. Within this family-reenactment, Ina pursues projects different from those of the therapist. The therapist's project is aimed at protecting the baby; however, Ina's project's aims have not yet fully materialized. The two of them make reference to an unconventional narrative they had developed the previous day, namely that the baby figure had chosen its parents. Ina and the therapist then give names to the figures they are forming: a family with mother, father and grandmother. A “thief” is also discussed, a figure that played a role in the previous day's session and will again do so in the following segments. Ina then asks for the therapist's help to form a new baby figure. They discuss which color the figure should have. After a short misunderstanding, Ina chooses blue. Despite indications that they are still pursuing diverging projects, in agreeing to form the baby together they share an empathic moment in “odd communion” with each other (Garfinkel, 1952, p. 114; Heritage, 2011b, p. 183), but their conflict is still unresolved.

### Segment 8—A Robber on the Stage

In the following segment, Ina and the therapist introduce a new character into the play activity. Within the play, this character is doing contrariness, which Ina and the therapist use as a stand-in to negotiate their actual conflict outside of the play.


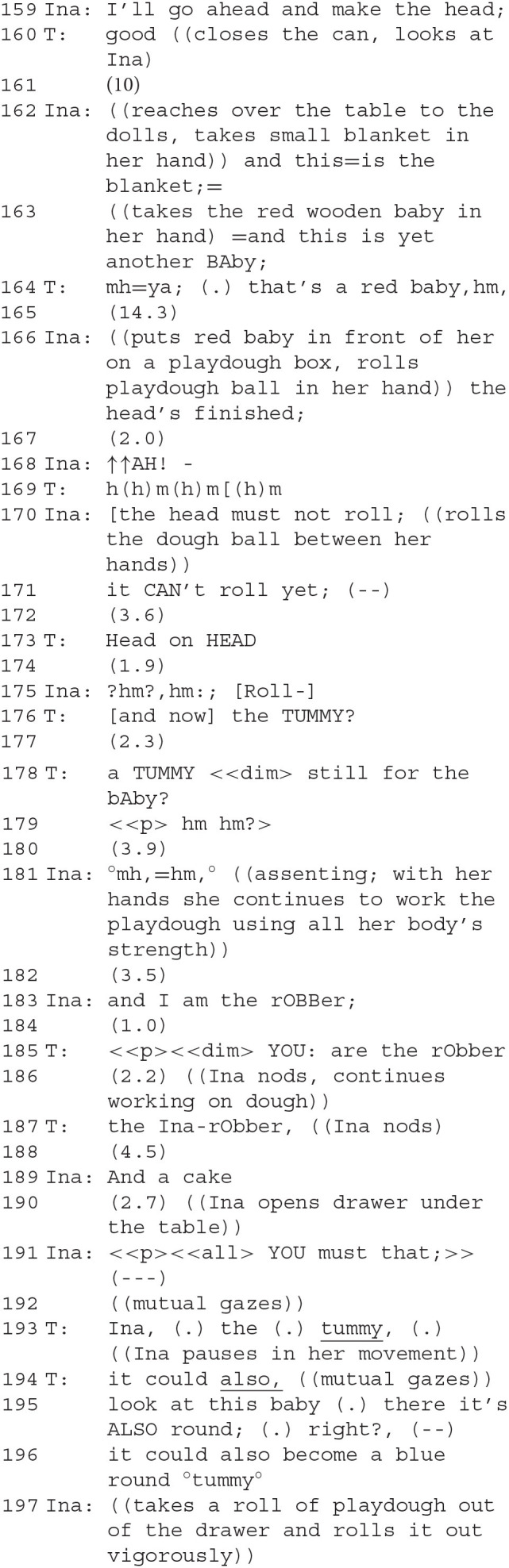


The therapist follows Ina in her project of forming another playdough baby. Ina comments on what she's doing with phrases like “this is…” (the blanket, still another baby, etc.). The therapist (164) seems to disagree when Ina produces another baby. Her utterance is prefaced with an extended “mh=ya: (.),” perhaps indicating the type of open class other-repair initiation that Corrin (2010) links to teaching situations between mother and child. The content of the therapist's statement that the baby is red borders on being superfluous (it is evident to all participants that it is red). The intention seems to lie in the implicature that it is *not blue*, and therefore going against what had earlier been agreed upon. She seems to perform this contrast-by-implicature *instead* of a more overt move pointing out the transgression against agreement, an instance of avoiding doing contrariness. Ina continues with verbal pointing: “the head's finished” (166). Throughout, the two share longer pauses while silently working, both are intensely engaged with building the stage and forming the baby. Ina's slightly alarmed warning that the baby's “head must not roll” is accompanied by the therapist's humming (169), which is apparently intended to calm Ina, who follows it up with “it can't roll” (171). Ina's gesture (right hand above her own head) is commented on by the therapist (173).

The therapist's proposal to form a tummy for the baby (176–181) is repeated and, after pauses, agreed to. After working silently for a while, Ina comes up with a surprising new play role for herself (183). A “thief” (german *Dieb*) had been mentioned by the therapist before (95). Now, a somewhat stronger term is used: a robber (german *Räuber*). This new character on the stage can be ascribed several meanings.

A first one is that Ina's taking up the role of the robber is a form of doing contrariness against the therapist. This view is supported by the subsequent interaction: the therapist uses a contrastive accent on the pronoun in her response, “You: are the robber” (185), evoking a comparison with other identities of the robber that she perhaps would have thought more plausible. The move is thus unexpected and gives Ina an initiative; it also affects their affiliative relationship because Ina's choice to take on the role of the robber, who is antagonistic toward the other figures, also sets the two players up as antagonists. Ina here exploits the porous boundary between her “actual” and her play role. Ina is outside of the play, the robber inside, but the two are the same person. The situation is reminiscent of topological objects such as a Klein bottle that have no clearly defined inside or outside^4^. Once again, as in the first and second segments, DC is practiced with an aspect of roleplay. The therapist adds a second meaning after a pause with a remark: “The Ina-robber” (187). Ina nods her agreement. In German, “der Inaräuber” is ambiguous between one who has stolen Ina and a robber named Ina. The therapist offers this new meaning which integrates active and passive aspects of this new character. The robber is the antagonist of the baby (we will see this unfold in the following sequences), but the therapist, instead of highlighting this contrast, makes an effort at *avoiding “*doing contrariness” by integrating the two contrasting accounts. She creates a “*conceptual* framework” (Goodwin, 2018) which keeps both the robbing subject-agent and the passively robbed child active.

From a clinical perspective, a third layer of meaning could be offered *via* biographical interpretation here: Ina sees herself as “robbed” from her biological parents by her adoptive parents. The robber is an “identification with the aggressor”; which is why Ina came into treatment. In this line of reasoning, Ina's fast transformation is a kind of re-telling of her biographical story, using the treatment for just the purpose of leaving the straightjacket of her history (Gallagher, 2015).

### Segment 9—A Scary Thief Appears

In the following segment contrariness escalates into violence. The therapist does not commit to a role either within *or* outside the play. The conflict is resolved by achieving mutual agreement to separate play roles from real roles.


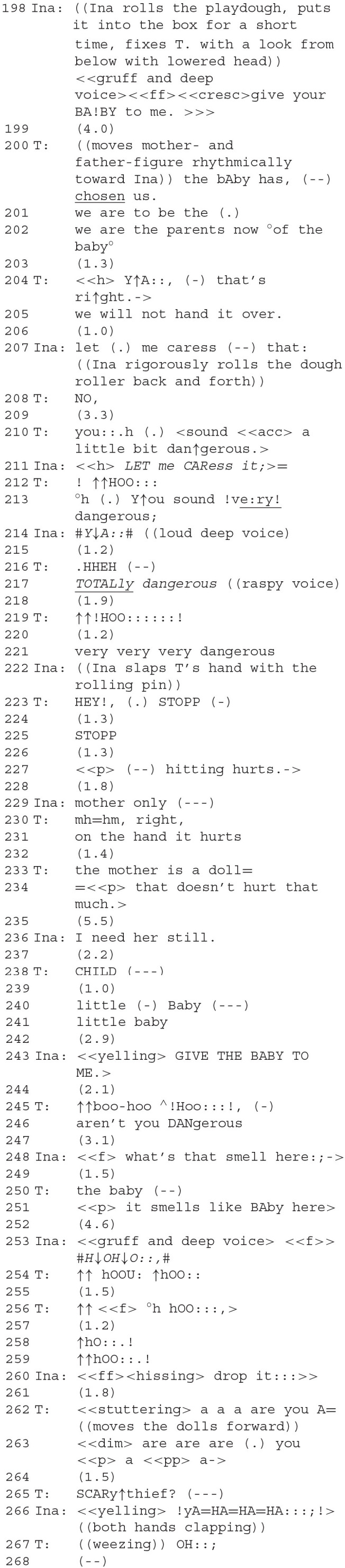


Ina turns into a robber with a loud and gruff voice; the therapist takes on the role of the adoptive parents who defend their custody of the baby (198–205). “Normal” family circumstances do not hold: the baby is said to have “chosen” the parents here (200). A strong antagonism between the two play roles is built up, initiated by Ina's adoption of the robber as play role, and furthered by the therapist's choice: since it was the adoptive parents who actually chose Ina, the therapist takes on a risky role—in Ina's eyes the therapist-as-adoptive-parent robbed the baby from her biological mother. Ina's antagonistic stance as the robber, adopted when she took on that role in an act of doing contrariness, is met in kind by the therapist. In the logic of the play world, the baby figure on the stage is actually situated between two robbers: a contrastive framework emerges with Ina on the one hand projecting to rob *and* to caress the baby; on the therapist's side the project is to protect *and* to adopt the baby, which is viewed by Ina as both “robbing” and “being robbed.”

To Ina's repeated and intensifying demands that she hand over the baby, the therapist-as-parent responds at first with a refusal and then by saying that Ina sounds dangerous, with each increase in vocal intensity of the demand mirrored by an increase in the dangerousness asserted by the therapist's responses (207–221). The conflict escalates when Ina hits the therapist's hand with the rolling pin, which she responds to by exclaiming “stop,” followed by a justification: “hitting hurts” (222f.). “Doing contrariness” has escalated into a small event of violence. The invisible border between the play world and the “real” world is torn apart for a moment. It is possible that Ina's hitting was performed also as an in-game action (this is implied by her justification, see below), but the therapist's reaction, the hand having been hit being that of both her in-play character and herself, is not.

As is so often the case, this escalation into violence is the culmination of a series of failed attempts at communicating. In the preceding turns, reiterations of Ina's demands followed by the therapist's commenting on how dangerous Ina sounds, make for somewhat anomalous adjacency pairs: normally, a provocation-response sequence of turns deals with a single issue, the issue is then either resolved or participants agree that they cannot agree (Farkas and Bruce, 2010). In either case, conversation moves on. This is clearly not the case here: conversation has come to a standstill; instead of progressing, the issue is reiterated with increasing intensity by both participants until it turns into an outburst of violence, a clear case of communication failure. We think that one reason for this failure might lie in how the therapist chooses to respond to Ina's demands: commenting on her dangerousness is not the response of a parent who is threatened by a robber, but that of an adult not fully entering into the play world of a child. By not committing fully to either role, the therapist robs Ina of a possibility to develop her play's progression: the therapist's responses, because they are given from an outside role, cannot be adequately reacted to from within the realm of Ina's play. As a child, Ina probably is still lacking the competence to switch between roles as effortlessly as the therapist. Thus, her only available response to the (from her perspective) illicit contributions of the therapist is to reiterate her own contributions, and to intensify them. The culmination into violence is here also preceded by both of them doing contrariness unchecked, with no attempt at repair being made.

The border between the worlds of play and reality is restored when Ina states that her hitting is aimed at the play mother, “mother only” (229), and the therapist accepts this, both agreeing that the mother doll might be hit but not the real hand of the therapist. This is a cooperatively executed repair, initiated by the therapist's exclamation to stop, but the solution for how it can be mended is first proposed by Ina and then accepted and implemented by both after the rules have been made explicit by the therapist. Previous attempts at resolving contrariness were failures perhaps also because they were unilateral. The two of them keep their antagonism to the world of the play (differentiating more properly between the two worlds) and thus do not allow it to affect the affiliation between the actual players.

We would like to argue that this complex act of conflict resolution is very similar to a repair: there is a deviant move, Ina's hitting, which causes the therapist to bring the previous course of interaction to a standstill, other-initiating the repair-like process (223–225). She then points out what was objectionable about Ina's move (“hitting hurts,” 227), targeting it as something to be remedied, a reparandum. Ina responds with a turn that is like a reformulation and a self-repair (“mother only,” 229) in the sense that it accepts the objection and clarifies that the object of her troublesome move was the mother figure, not the therapist. The therapist seems to accept this (“right,” 230) as an agreement that hitting should be restricted to the play figures (231–234), and conversation is then allowed to continue. The trouble-source here is a transgression against social norms, but dealing with such can be treated as an instance of repair (Albert and Ruiter, 2018). Ina initiates conversation again after a pause (236): “I need her still.” It is clear that by “her,” Ina refers to the “mother” figure which they had just agreed could be hit instead of the therapist (233). This is ambiguous between Ina saying that she still needs the mother figure for playing later, or that she is in need of a real mother. After another pause, the therapist responds with a mother's calls for her child (238–242), suggesting that she adopts the latter interpretation. However, Ina responds by yelling loudly and demanding to be given the baby as the robber (243): they are back in the play world. After this suspension of antagonism between their actual world roles, they take their doing contrariness up again, now perhaps limited to their play roles.

For the sake of brevity, we leave out some aspects of what happens next, but note that after the therapist plays a fearful mother (imitating a scared stutter), she finds a new name for Ina's play role: “a scary thief” (265), which Ina enthusiastically confirms.

CA studies of adult therapy draw attention to therapeutic (re-)formulations. A consistent result is that therapists use reformulations in order to turn a patient's attention to special moments of expression (Antaki, 2008) or to help a patient find the right word for an experience (Rae, 2008). This is what the therapist tries to do here, by commenting on and increasing the “dangerous” aspect of Ina's utterances. The therapist's action is best described by what (Deppermann, 2011) calls a “nationalization;” although her attempts seem to fail initially leading to the violent outburst (210–225), once she calls Ina's play role a “scary thief,” Ina can agree. Finding an (unconventional)^5^ term that matches an unclear idea, nailing down something foggily imagined, or defining a role with a name seems to be a helpful strategy in adult (Knol et al., 2020) and child therapy. It collects a multitude of individual experiences into a coherent category. As in Deppermann's examples, a pause (264) precedes the notionalization, possibly indicating a cognitive process of word finding in the therapist's mind. The notionalization is delivered here by imitating the low pitch of Ina's voice and is thus introduced “in-play” instead of as an outside labeling, an effort aimed again at *avoiding* contrariness in this essential therapeutic act. The notionalization encapsulates in a single word how Ina feels scared and how she—in her role as robber—scares others.

### Segment 10—A Baby Thrown in the Compost

Ina achieves a moment of reconciliation with her devalued baby-self.


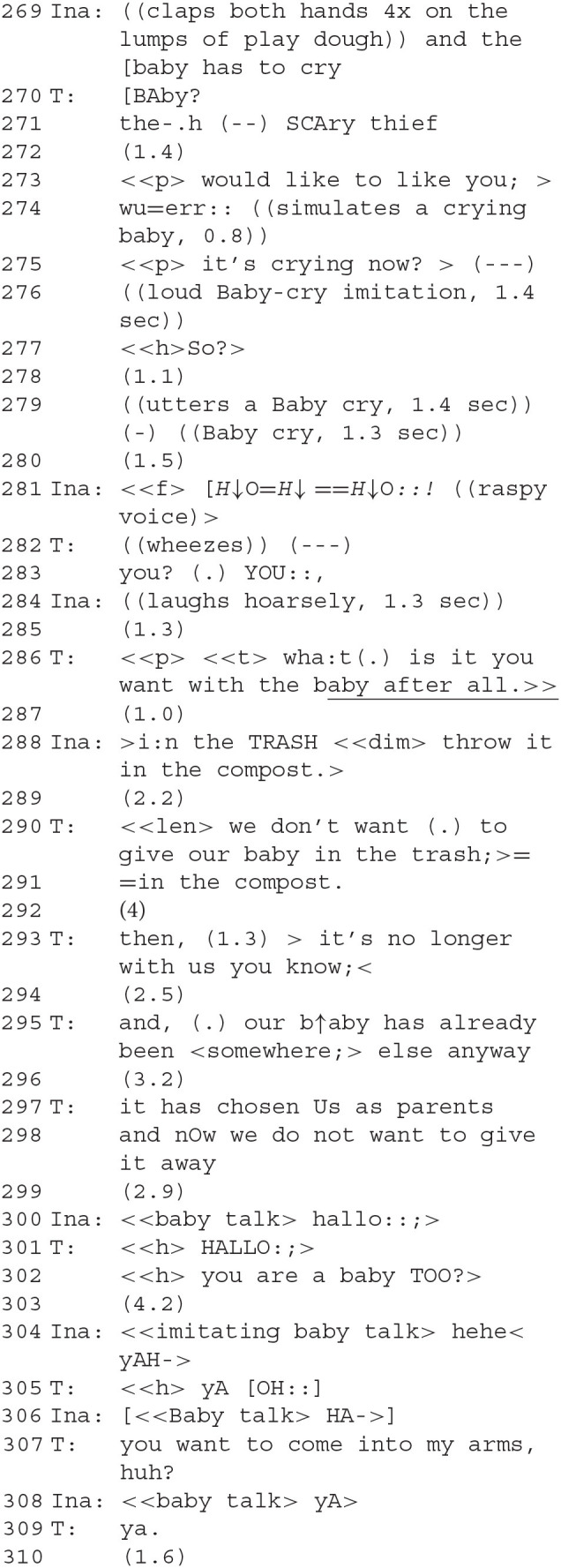


The conversation is accompanied by strong performance sounds. The scary thief is presented very powerfully, expressing dominance rhythmically, bodily, and vocally. The therapist's question about what the scary thief “wants with the baby after all” (286) is answered in a surprising fashion: it is to be thrown on the compost.

Therapeutically, this is a crucial and emotionally touching scene. We have seen how the thief/robber and the baby took sharply antagonistic roles and how difficult it was for Ina to find an exit from a biographical dilemma—does she want to be the baby or to have it? Did she choose her parents or was she chosen? A radical solution to the increasing contrariness and antagonisms is to get rid of the baby as if it were trash. The therapist works against this solution. Her play-parent voice says she does not want to give the baby away (290 f.). However, after some reasoning by the therapist, a new interaction starts unexpectedly (300): Ina uses a baby-like voice to utter a greeting, and the therapist responds by regreeting and asking if Ina is now “also a baby.” The question whether the voice, which had been the scary thief a moment ago, is “also a baby” is answered twice: first, as a direct answer to the question, “hehe yeah” (304) after a pause. Then a second time, when the therapist invites her to be picked up, and she agrees (307–308). As the scary thief, Ina despised the baby role, but she accepts it for herself now. In a sense, she repairs the affiliation to her own baby-self which she had opposed before by acting against it as the robber (and already in a milder form in segment 2). This only lasts for a moment, however.

### Segment 11—A Baby Found on Grandma's Arm

The moment of reconciliation passes again. However, both find a way to provide Ina with solace after her traumatic biographical experience is reunderstood. A baby given away by her mother can still find affection from her grandmother or from her therapist. An “odd communion” is created.


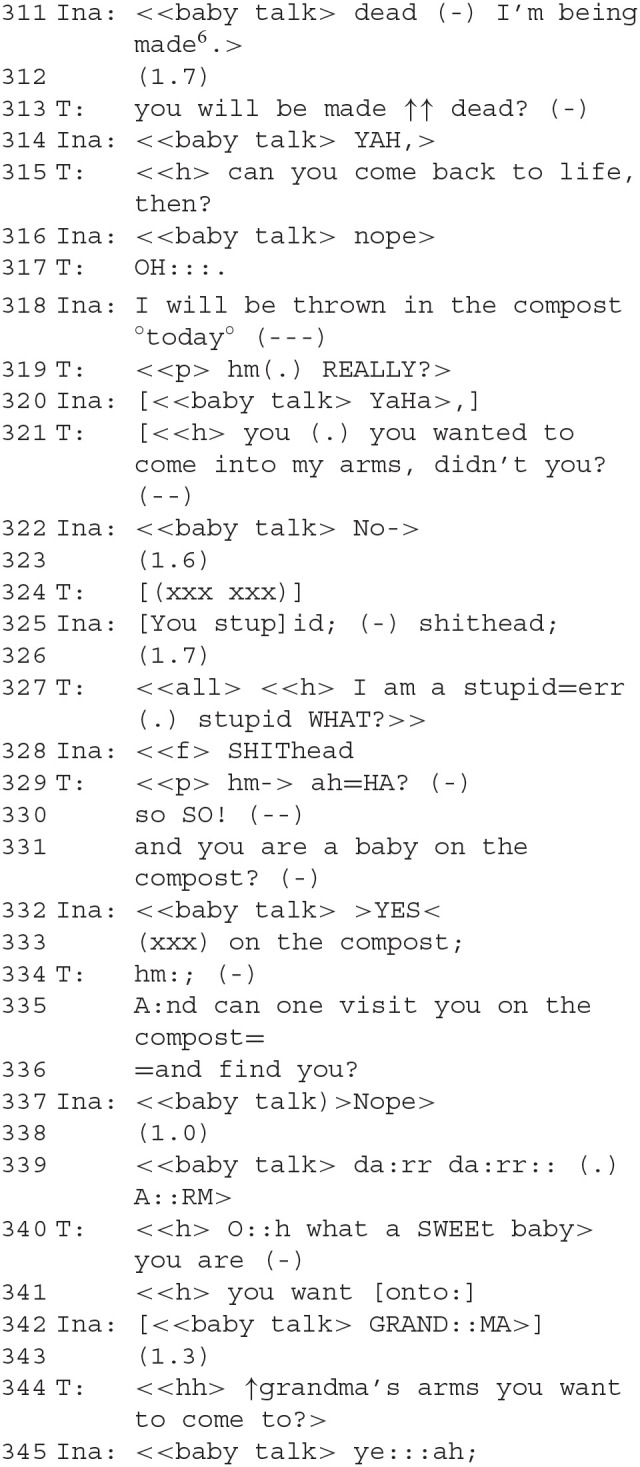


Ina develops a full phantasmatic narrative: the baby will be “made dead” (311) and is thrown on the compost “today” (318). The therapist responds with utterances of incredulous amazement. Ina finds a way out of her life narrative: the adoptive parents have robbed the baby away from the natural parents in Ina's view. A fight unfolds in the play. The therapist, on the side of her adoptive parents in Ina's view, is insulted as a “stupid shithead” (325, 328). “Doing contrariness” is increased up to this verbal violence. The baby is to be thrown on the compost. However, the therapist now replies to the insult mildly with acknowledgment tokens [“mh ah=HA” and “soso!,” (329)], instead of opposing this solution. By the therapist accepting Ina's assertion of being on the compost (331) in a calm voice, she trades her claim for epistemic and deontic authority about the state of affairs for a renewal of their affiliation (characteristics of ADC), so that they both meet at the same emotional height at the same place. Ina now responds calmly to the therapist's confirmation question that she is a baby on the compost, namely with a clearly pronounced “YES” repeating “on the compost” (333). This is the place where she situates her baby-self. Because the two have accepted this set of affairs for the time being and are no longer in conflict about it, an “odd communion” in Garfinkel's terms has been set up there, a common order of a “strange community” that can be lived by those involved—on a compost heap or in treatment rooms.

“Under such circumstances, the practical achievement of an empathic moment concerns, to adapt Garfinkel's (1952, p. 114) marvelous phrasing, how persons ‘isolated, yet simultaneously in an odd communion, go about the business of constructing an order together.” (Heritage, 2011b, p. 183)

From clinical experience (Vischer and Vischer, 1987), it is known that to be in the arms of adoptive parents would be perceived as something like a betrayal of the biological mother. Grandmothers are often more easily accepted by adoptive children. A similar dynamic seems to be in place here as well: by calling for a grandmother in babytalk (342), Ina initiates a role change by the therapist. This solution is a repair to the previously failed attempts (at 307 and again at 321) at providing Ina with the solace of an embrace despite the contrariness in the roles they were playing; the therapist picks this up (344) and *via* the role-change they can restore their emotional affiliation and comfort Ina. The conflict is not resolved but suspended and delegated to other roles for the time being. Once again, a role change is cooperatively constructed as part of a complex repair allowing for an affiliative restoration.

## Discussion and Conclusion

Securing emotional affiliation is considered to be one of the main tasks in psychotherapeutic treatment, with adults and more so with children. “Empathy” is a concept in psychotherapy process research (Elliott et al., 2018) that has been often studied but not yet fully understood (Weiste and Peräkylä, 2014; Buchholz et al., 2017). CA has identified “challenge” as the counterpart to empathy (Voutilainen et al., 2018). Empathy and challenge are concepts informing conversational practices used by therapists in order to achieve change. Clinicians (Giora, 1989) know that children sometimes are a challenge for adults, and we have learned that adult patients sometimes display high levels of empathy for their therapists (Dekeyser et al., 2009; Buchholz and Kächele, 2015, 2017; Buchholz et al., 2015). We focused on the complex practice we call “doing contrariness,” and two ways to deal with it, the pre-emptive strategy “avoiding contrariness,” and “remedying contrariness,” a kind of repair. In our analysis of this session, we used them as concepts that could describe conversational practices used by both participants.

In our analysis, “Doing contrariness” was observed in different modes: *via silence* (refusing goodbye as “noticeable absence” which provoked repair activities); as an *embodied* practice (prosodically, using voice and other parts of the body); and as a practice of conflicting conversational *projects*. These modes have one feature in common: violation of (communicative and social) expectations, at the cost of risking affiliation and/or epistemic agreement. Which mode will be used depends on situated opportunities. We have seen it here often sequentially practiced after a delay and responded to with a pause, which aligns with previous findings about the correlation of delay in responses and their unexpectedness (Bögels et al., 2015; Kendrick and Torreira, 2015). Both might indicate a cognitive calculation of risks related to violating expectations and of how to respond. In the beginning, we broadly characterized “doing contrariness.” We observe two recurrent elements: (a) communicative behavior (including the omission of actions) that goes against the conventional expectations for the interaction situation and its participants (dictated in part by the maxims of cooperative communication (Grice, 1989) and in part by sociocultural norms); (b) the relation between interlocutor affiliation and claim to authority, which often turns out to be an exchange equation. This relation is affected by this communicative behavior. We have seen that it can differentially target affiliations projected by several roles an interlocutor might assume, i.e., it is highly sensitive to the social and interactional positioning of interlocutors. Its disruptive effect on affiliation is such that it can actively change participative frameworks by excluding some members and that it can contribute to an escalation into violence. Some aspects of “doing contrariness” can be probably described formally in terms of “strategic conversation” (Asher and Lascarides, 2013), others *via* the more socioculturally oriented concept of “impoliteness” (Culpeper, 1996, 2011; Bousfield, 2010).

We suggest in addition that it would be extremely useful for the interactional analysis not only of psychotherapy to develop general criteria for when turn sequences can be considered anomalous or deviant and to study how this relates to non-progression of communication or even violent outbursts.

We have also seen that aspects of how to deal with “doing contrariness” can be considered part of a larger typology of practices including repairs, especially if a definition of repair is employed that also allows it to target transgressions against social conventions (Albert and Ruiter, 2018). “Doing contrariness” often constitutes a claim to authority insofar as a participant uses it to diverge from a cooperatively followed conversational path and instead obliges their interlocutors to adapt to a unilaterally executed move (e.g., Ina's silence, her hitting). This normally effects a disaffiliation between interlocutors unless their power imbalance is such that such unilateral moves are allowed for one of the participants. On the other hand, “avoiding” contrariness can meaning to forgo an authoritative claim (e.g., a correction) that could be made for the sake of maintaining or strengthening affiliation, as seen in segment 3. In our analysis, we have observed several instances of attempts at “avoiding” and “remedying” it, both successful and unsuccessful. “Avoiding” it means a pre-emptive self-initiated attempt at reducing the negative effects of a DC-move on both the conversation and the affiliation, or to perform a different move instead of DC. “Remedying” DC is a kind of complex repair. Notably, the most successful attempts at remedying contrariness we observed consisted of collaborative efforts, in which both participants had to signal their agreement to accept a proposed solution as a repair. We suggest that such a solution involves an additional step in learning and distinguishing: in a further unexpected move, participants conducted a shift from affiliation to epistemics or between roles they assumed and thus opened a new way for re-establishing communication. RDC can be related to the taxonomy of repairs developed by Albert and Ruiter (2018). They describe the category of “other-initiated other repair” (OIOR), where a reparandum in the speaker's talk is both identified and repaired by the interlocutor, and state that this is far rarer and more difficult compared with self-repair. As they (p. 296–298) point out, forms of OIOR are frequently accompanied by acts professing hesitation or reluctance, and in everyday interaction found more regularly directed against children than against adults. Self-repair as a conversational practice is a skill children gradually learn in the course of development (Forrester, 2008). In our case study, we have seen RDC as other-oriented (segments 1 and 8) as well as self-oriented ADC (segment 3), and we have distinguished a third kind, that of cooperatively executed repair (segments 8 and 11). Probably because dealing with contrariness targets affiliative relationships more than conversational fluency, this last type seems to be the most successful because it necessarily involves willing cooperation between participants.

Doing and resolving contrariness affects the collaborative effort at discourse progression which requires “that the separate perceptual frameworks of each participant must be integrated into a common task” (Goodwin, 2018, p. 295), here that of doing child psychotherapy. We think that the concepts of doing/resolving contrariness can also be related to the concept of (the repair of) therapeutic alliance ruptures (Safran et al., 2011). While DC/ADC describe events at the very local scale of conversation, such events have the potential to lead to a perceived rupture in the alliance between therapist and patient, a “tension or breakdown in [their] collaborative relationship” (Safran et al., 2011, p. 80), at the scale of their (current) overall relation. The concept of the alliance and suggestions for how to repair its ruptures are based on adult therapy, where the therapeutic task is much more transparently discussable than in child therapy. For future research, it would be worthwhile to investigate under what circumstances DC/RDC/ADC events can lead to or prevent such ruptures, both in adult and in child therapy.

As a last discussion point, there is also another concept that is something of a counterpart to “doing contrariness.” It is “doing vulnerability”:

“If both interaction and individual are autonomous systems, then they are in continual tension with each other in each ongoing interaction. These tensions get manifested in what might be called vulnerability. What is interesting about the confluence of enaction and interactional sociology that we propose in this paper, is that both the individual and the interaction can be conceptualized as vulnerable. Vulnerability hangs together closely with autonomy. It is at the interplay between individual and interactional autonomy and vulnerabilities that the co-creation of significance and significant action happens ….” (Jaegher et al., 2016, p. 6)

Applying CA and other methods of interactional analysis to psychotherapeutic processes requires considering how to approach vulnerability as the center of meaning making. It is undeniable that Ina showed her vulnerability as the downside of “doing contrariness,” and that both the therapist and her father (in segment 1) make efforts at protecting it. Her vulnerability has a history in very early life, and it produced effects that rendered her nearly incapable of accepting a kind of support and help which her young mind understood as betrayal. The practice of child therapy should not be analyzed without a profound understanding of such traumatic experiences and of psychological development. We have tried to integrate some insights from these domains. To fully integrate trauma and vulnerability into CA, studies of psychotherapy will be a task for the future.

## German Transcripts

If readers are interested in the original German transcripts—please turn to the first author.

**Contact:** Prof. Dr. Michael B. Buchholz, Dipl.-Psych., social scientist, psychoanalyst, Prof. (em.) for Social psychology at International Psychoanalytic University (IPU) Berlin (Germany). michael.buchholz@ipu-berlin.de.

## Data Availability Statement

The original contributions presented in the study are included in the article/supplementary material, further inquiries can be directed to the corresponding author/s.

## Ethics Statement

Written informed consent was obtained from the individual(s), and minor(s)' legal guardian/next of kin, for the publication of any potentially identifiable images or data included in this article.

## Author Contributions

BW conducted the therapeutic session providing the primary data for the study. MB conceived of the general idea of the study. MB, TB, and BW contributed to the analysis. MB wrote the first draft and TB revised draft of the paper together with MB.

## Conflict of Interest

The authors declare that the research was conducted in the absence of any commercial or financial relationships that could be construed as a potential conflict of interest.

## Footnotes

^1^“…interactional practices developed for ordinary conversational contexts can have dysfunctional consequences when they are unreflectingly implemented within the medical visit” (Heritage, 2011a, p. 339).

^2^Readers who wish to study the German transcripts should contact the first author.

^3^The line and segment numbering is left as it is in the German original transcript, to facilitate comparison. If you are interested in the original transcript, please contact the first author.

^4^Conversation analysts have observed such topological, Moebius-like, confusion in analyzing talk-in-interaction: see, e.g., Garfinkel and Rawls (2016, p. 145); Jefferson et al. (2015, p. 143).

^5^In German, it is “Gruseldieb,” a compound coined *ad hoc*.
